# The *Neurospora crassa* Standard Oak Ridge Background Exhibits Atypically Efficient Meiotic Silencing by Unpaired DNA

**DOI:** 10.1534/g3.119.400006

**Published:** 2019-03-06

**Authors:** Dev Ashish Giri, Ajith V. Pankajam, Koodali T. Nishant, Durgadas P. Kasbekar

**Affiliations:** * Centre for DNA Fingerprinting and Diagnostics, Hyderabad 500039, India; † Graduate Studies, Manipal University, Manipal 576104, and; ‡ School of Biology; § Centre for Computation Modelling and Simulation, Indian Institute of Science Education and Research, Thiruvananthapuram 695551, India

**Keywords:** chromosome segment duplication, genome heterozygosity, introgression, RIP suppression

## Abstract

Meiotic silencing by unpaired DNA (MSUD), an RNAi-mediated gene silencing process, is efficient in crosses made in the *Neurospora crassa* standard Oak Ridge (OR) genetic background. However, MSUD was decidedly less efficient when the OR-derived MSUD testers were crossed with many wild-isolated strains (W), suggesting that either sequence heterozygosity in *tester* x W crosses suppresses MSUD, or that OR represents the MSUD-conducive extreme in the range of genetic variation in MSUD efficiency. Our results support the latter model. MSUD was less efficient in near-isogenic crosses made in the novel *N. crassa* B/S1 genetic background, and in *N. tetrasperma* strain 85. Possibly, in B/S1 and 85, additional regulatory cues, absent from OR, calibrate the MSUD response. A locus in distal chromosome 1R appears to underlie the OR *vs.* B/S1 difference. Repeat-induced point mutation (RIP) destroys duplicated genes by G:C to A:T mutation of duplicated DNA sequences. Chromosome segment duplications (*Dp*s) dominantly suppress RIP, possibly by titrating out the RIP machinery. In *Dp* x *N* crosses, the *Dp*–borne genes cannot pair properly, hence efficient MSUD, as in OR, silences them and renders the crosses barren. We speculate that the increased productivity engendered by inefficient MSUD enables small duplications to escape RIP.

## Introduction

Meiotic silencing by unpaired DNA (MSUD) was discovered in crosses made in the standard Oak Ridge (OR) genetic background of *Neurospora crassa* (Aramayo and Metzenberg 1996; Shiu *et al.* 2001; Perkins 2004). Any gene not properly paired with its homologous sequence during meiosis is transcribed into ‘aberrant RNA’ that is then processed into single-stranded “MSUD-associated small interfering RNA” (masiRNA), which directs a silencing complex to degrade complementary mRNA, and thus silences the unpaired gene and any other genes homologous to it (Hammond *et al.* 2013b; Hammond 2017). The ::*act*, ::*asm-1*, ::*Bml^r^*, ::*mei-3*, and ::*r^+^* MSUD tester strains contain an additional copy of the *act* (*actin*), *asm-1^+^* (*ascospore maturation-1*), *Bml* (*β−tubulin*), *mei-3*, or *r^+^* (*round ascospores*) gene inserted at an ectopic location. The ectopic copy is unpaired in a *tester* x OR cross and induces the production of masiRNA which degrades its complementary mRNA, and the resulting deficit of actin, ASM-1, β-tubulin, MEI-3, or R protein manifests as striking ascus or ascospore phenotypes, whereas in a homozygous *tester A* x *tester a* cross the ectopic copy is paired, therefore there is no MSUD, and ascus and ascospore development is normal (Raju *et al.* 2007).

Surprisingly, MSUD was not as efficient when the OR-derived tester strains (*tester^OR^*) were crossed with several wild-isolated *N. crassa* strains (Ramakrishnan *et al.* 2011). Of 80 wild-isolated strains tested in crosses with the ::*Bml^r^* and ::*mei-3* testers, only eight, designated as the “OR” type, showed silencing phenotypes comparable to the *tester^OR^* x OR crosses. Crosses with four wild strains designated as the “Sad” type did not show MSUD, and the remaining 68 strains showed an intermediate phenotype, in that, the crosses silenced *bml* but not *mei-3*^+^, and they were designated the “Esm” type. One hypothesis (model 1) to explain these results posits that sequence heterozygosity between the *tester^OR^* and wild genomes either overwhelms the MSUD machinery or causes asynapses and self-silencing of one or more MSUD gene. This model extends the observations that deletion alleles of several MSUD genes can act as semi-dominant suppressors of MSUD (Samarajeewa *et al.* 2017 and references therein), presumably because they cause their wild-type homolog to be unpaired, induce its autogenous silencing, and thus “silence the silencer” (Hammond 2017); and that a 6% divergence over 4.5 kb between the *r^+^* and *r^RIP93^* alleles could induce silencing of the *r^+^* allele (Pratt *et al.* 2004). The *sad-1Δ* and *sad-2Δ* deletions (*i.e.*, *Sad-1* and *Sad-2* (*Suppressor of ascus dominance-1* and *-2*)) were strong dominant suppressors whereas the other MSUD gene deletions were less effective, possibly because of high expression or long protein half-life (Hammond *et al.* 2013a; Decker *et al.* 2017). Shiu and Metzenberg (2002) also generated *Sad-1* strong alleles by the RIP (repeat-induced point mutation) process, which showed that if the *sad-1* gene sequence is sufficiently altered then the *sad-1^+^* allele can become unpaired and induce its autogenous silencing, thus supporting the idea that sequence heterozygosity can promote gene silencing. The *Sad-1* and *Sad-2* dominant MSUD suppressors suppressed the classical ascus-dominant mutants *Ban* (*Banana*), *Dip-1* (*Diploid ascospores-1*), *Pk*^D^ (*Peak-Dominant*), and *R* (*Round ascospores*), in which known or suspected deletion alleles induce MSUD in their wild-type counterparts (*ban^+^*, *dip-1^+^*, *pk^+^*, and *r^+^*). The *Sad-1* and *Sad-2* suppressors also suppressed the barren phenotype of crosses heterozygous for chromosome segment duplications (*i.e.*, *Dp* x *N*), suggesting that the barrenness is caused by MSUD (Kasbekar 2013; Shiu *et al.* 2001; 2006). *Dp(EB4)* and *Dp(IBj5)* strains contain duplications of, respectively, 35- and 115-gene segments (Kasbekar 2013). Crosses of *Dp(EB4)* and *Dp(IBj5)* strains with the OR type wild strains were barren, with the Sad type were fertile, and with the Esm type were, respectively, fertile and barren (Ramakrishnan *et al.* 2011). Reduced MSUD in the related species *N. tetrasperma* also was attributed to asynapsis and silencing of the *sad-1* gene because of structural difference between the mating-type chromosomes (Jacobson *et al.* 2008).

An alternative hypothesis (model 2) posits that natural populations harbor a wide variation in MSUD strength, and the OR strains represent the MSUD-conducive extreme. Model 1 predicts that crosses isogenic for a wild-isolated Sad type genetic background would show an OR-like MSUD phenotype. Here, we examined MSUD in tester-heterozygous crosses made in a novel isogenic background generated from the Sad type *N. crassa* wild strains Bichpuri-1 *a* and Spurger-3 *A*.

MSUD is also much less efficient in *N. tetrasperma* strain 85 (Jacobson *et al.* 2008; Ramakrishnan *et al.* 2011). Specifically, self-cross of an 85-derived [::*asm-1* + WT] dikaryotic strain produced < 76% MSUD-induced white-spored asci whereas comparable crosses in OR produced >99% white-spored asci (Ramakrishnan *et al.* 2011), thus 85 can be thought of as a Sad- or Esm-type background. We have now introgressed an ::*r* transgene from *N. crassa* OR into *N. tetrasperma* 85, and used the resulting ::*r^Nt^* testers to make ::*r^Nt^* x 85 crosses. We had previously introgressed the *N. crassa T(EB4)* and *T(IBj5)* translocations into *N. tetrasperma* and obtained *T(EB4)^Nt^*, *T(IBj5)^Nt^*, *Dp(EB4)^Nt^* and *Dp(IBj5)^Nt^* strains in which nominally only the rearrangement breakpoints were from *N. crassa* while the rest of the genome was from *N. tetrasperma* strain 85 (Giri *et al.* 2015). Since MSUD causes *Dp*-heterozygous crosses to become barren, and MSUD is less efficient in *N. tetrasperma* strain 85 than in *N. crassa* OR, we would expect *Dp*-heterozygous crosses in *N. tetrasperma* 85 to be more productive than their *N. crassa* OR counterparts. We used the *T^Nt^* and *Dp^Nt^* strains to test whether *Dp*- and *T*-heterozygous crosses were comparably productive in *N. tetrasperma*.

During an *N. crassa* or *N. tetrasperma* sexual cross, the parental *mat A* and *mat a* haploid nuclei fuse to produce a diploid zygote nucleus that undergoes meiosis and a post-meiotic mitosis to generate eight haploid progeny nuclei (4 *mat A* plus 4 *mat a*). In *N. crassa* these nuclei are partitioned into the eight initially uninucleate ascospores that form in an ascus, whereas *N. tetrasperma* asci form four larger initially binucleate ascospores, each receiving a *mat A* and *mat a* pair (Raju 1992; Raju and Perkins 1994). Consequently, the mycelium generated from an *N. crassa* ascospore is homokaryotic for mating type and requires mycelium from another ascospore of opposite mating type to complete the sexual cycle (heterothallic lifecycle), whereas the mycelium generated from a dikaryotic [*mat A* + *mat a*] *N. tetrasperma* ascospore contains both *mat A* and *mat a* nuclei and can undergo a self-cross (pseudohomothallic lifecycle). Dikaryotic mycelia can make some homokaryotic conidia (vegetative spores) by chance. Also, during *N. tetrasperma* ascogenesis, a pair of smaller homokaryotic ascospores can occasionally replace a dikaryotic ascospore to form a minor fraction of 5-8 spored asci. Mycelia from homokaryotic ascospores and conidia enable *N. tetrasperma* to out-cross with like mycelia of the opposite mating type. The *Eight spore* (*E*) mutant increases the replacement of dikaryotic ascospores by homokaryotic pairs, and *E* x *WT* crosses produce many 8-spored asci, although *E* x *E* crosses are infertile (Dodge 1939; Calhoun and Howe 1968). Does MSUD underlie the ascus-dominant *E* mutant phenotype? An earlier attempt to generate *N. tetrasperma Sad-1* mutants did not yield an allele that could induce silencing of its *sad-1^+^* homolog (Bhat *et al.* 2004), therefore here we have introgressed a strong *N. crassa Sad-2* MSUD suppressor into *N. tetrasperma* 85 and compared the *Sad-2* x *E* and *Sad-2^+^* x *E* crosses.

## Materials and methods

### Neurospora strains, culture methods, and crosses

Unless indicated otherwise, all Neurospora strains (Table S1) were obtained from the Fungal Genetics Stock Center (FGSC; McCluskey *et al.* 2010), Department of Plant Pathology, 4024 Throckmorton Plant Sciences Center, Kansas State University, Manhattan, KS 66506, USA. Neurospora was cultured essentially as described by Davis and De Serres (1970), using Metzenberg’s (2003) alternative recipe to make Vogel’s medium N. Crosses were made at 25° by simultaneously inoculating mycelial plugs of the parental strains on synthetic cross medium supplemented with 1% sucrose and 2% agar.

*N. crassa*: The standard laboratory Oak Ridge strains 74-OR23-1VA (FGSC 2489) and 74-ORS-6a (FGSC 4200) will be referred to henceforth as OR *A* and OR *a*. The wild-isolated strains Bichpuri-1 *a* (P750) and Spurger-3 *A* (FGSC 3201) were used to generate the novel B/S1 strains (see below). The RLM 30-12 strain (genotype *Sad-2* (*RIP32*) *A*) containing a RIP-mutated *Sad-2* allele was a gift from Robert L. Metzenberg and is described by Shiu *et al.* (2006). The strains ISU 3118 (genotype: *rid his3*; *mus-52*Δ::*bar*; *VIIL*::*r^ef1^-hph A*) and ISU 3119 (genotype *rid his-3*; *mus-52*Δ::*bar*; *VIIL*::*r^ef3^-hph A*) are OR-derived MSUD testers bearing the transgenes ::*r1* and ::*r3* and were a gift from Dr. Tom Hammond, Illinois State University, Normal, IL 61790, USA. They are described by Samarajeewa *et al.* (2014). The 5.5 kb long *r^+^* gene (ncu02764, nucleotides 9,280,963 to 9,286,536) on chromosome 1 encodes a 3.3 kb ORF that shows robust MSUD that is suppressible only by the strong *Sad-1* and *Sad-2* suppressors, unlike other genes (*act*, *asm-1*, *mei-3*) in which MSUD is suppressed by even the weaker MSUD suppressors (Hammond *et al.* 2013a). These testers are henceforth referred to as ::*r1^OR^* and ::*r3^OR^* to distinguish them from the ::*r1^B/S1^* and ::*r3^B/S1^* testers that we constructed in the B/S1 genetic background (described below). The insertion sites of the ::*r1^B/S1^* and ::*r3^B/S1^* transgenes are identical to those of ::*r1^OR^* and ::*r3^OR^*.

Strains #1 (genotype ::*r1^OR^a*) and #6 and #15 (genotype ::*r1^OR^A*) were obtained as segregants from the cross ISU 3118 x OR *a*, and strains #1 and #10 (genotype ::*r3^OR^a*), and #11 (genotype ::*r3^OR^A*) were obtained from ISU 3119 x OR *a*. Strains #4 (genotype ::*r1^B/S1^ a*) and #1 (genotype ::*r1^B/S1^ A*) were obtained from ::*r1^B/S1^ A* x B/S1 *a*, and strains #4 and #16 (genotype ::*r3^B/S1^A*) and #15 and #24 (genotype ::*r3^B/S1^ a*) were obtained from ::*r3^B/S1^ A* x B/S1 *a* (described below). The results of crosses made with the ::*r1^OR^* and ::*r1^B/S1^* strains are presented in Table S3, and those with ::*r3^OR^* and ::*r3^B/S1^* are presented in Table 1.

**Table 1:** Round ascospore fractions from crosses heterozygous or homozygous for an ::*r3* transgene

Serial No.	Cross	N (x10^5^)	Round ascospores (%)
1	OR *a* x OR *A*	2.9	0
2	::*r3^OR^ a* x OR *A*	1.9	96.9 ± 0.8
3	OR *a* x ::*r3^OR^ A*	2.9	99.4 ± 0.3
4	::*r3^OR^ a* x ::*r3^OR^ A*	2.4	1.0 ± 0.2
5	B/S1 *a* x B/S1 *A*	0.4	0
6	::*r3^B/S1^ a* x B/S1 *A*	0.2	23.4 ± 2.5
7	B/S1 *a* x ::*r3^B/S1^ A*	0.2	22.3 ± 1.8
8 (i)	::*r3^B/S1^ a* x ::*r3^B/S1^ A*	0.001	7.5
8 (ii)	::*r3^B/S1^ a* x ::*r3^B/S1^ A*	0.006	5.7
9	B/S1 *a* x OR *A*	6.4	0
10	::*r3^B/S1^ a* x OR *A*	0.9	25.8 ± 2.9
11	B/S1 *a* x ::*r3^OR^ A*	3.3	38.0 ± 2.0
12	::*r3^B/S1^ a* x ::*r3^OR^ A*	1.1	3.8 ± 1.0
13	OR *a* x B/S1 *A*	1.7	0
14 (i)	::*r3^OR^ a* x B/S1 *A*	0.8	63.9 ± 1.3
14 (ii)	::*r3^OR^ a* x B/S1 *A*	1.8	59.0 ± 2.15
15 (i)	OR *a* x ::*r3^B/S1^ A*	1.5	43.8 ± 2.3
15 (ii)	OR *a* x ::*r3^B/S1^ A*	2.0	41.7 ± 0.7
16	::*r3^OR^ a* x ::*r3^B/S1^ A*	0.9	2.4 ± 0.4

N = number of ascospores harvested. Round ascospore fractions are given as mean percentage ± SEM from three technical replicates of each cross. Crosses in the OR background were harvested on days 21-25, and the other crosses on days 26-31. For ::*r3^OR^ a* x B/S1 *A*, ::*r3^B/S1^ A* x OR *a*, and ::*r3^B/S1^A* x ::*r3^B/S1^ a* two biological replicates were performed with different ::*r3* strains (respectively, #1 and # 10, and # 4 and # 16, and the ::*r3^B/S1^* homozygous crosses were #24 x #16 and #15 x #4).

*N. tetrasperma*: The reference strains 85 *A* (FGSC 1270) and 85 *a* (FGSC 1271); the *E* mutants *lwn*; *al(102)*, *E A* (FGSC 2783) and *lwn*; *al(102)*, *E a* (FGSC 2784) (hereafter *E A* and *E a*). Previously, we had introgressed the *N. crassa T(EB4)* and *T(IBj5)* translocations into *N. tetrasperma* to construct the strains *T(EB4)^Nt^* and *T(IBj5)^Nt^*, and from the *T^Nt^* x 85 crosses we obtained the self-fertile dikaryotic strains [*T^Nt^* + *N*] and [*Dp^Nt^* + *Df^Nt^*] (Giri *et al.* 2015). Homokaryotic conidial derivatives of genotype *T(EB4)^Nt^ a*, *T(IBj5)^Nt^ a*, *Dp(EB4)^Nt^ A* and *Dp(IBj5)^Nt^ a* were obtained from these dikaryons.

The *Sad-2^Nt^* and ::*r3^Nt^* strains were made by introgressing the relevant *N. crassa* gene into the strain 85 genetic background (see below). They were used to measure MSUD efficiency in ::*r3^Nt^*-heterozygous and ::*r3^Nt^*-homozygous crosses and in ::*r3^Nt^* x *Sad-2^Nt^*. The superscript “*Nt*“ in the strain designations (eg., ::*r3^Nt^ A*, ::*r3^Nt^ a*, *Sad-2^Nt^ A*, and *WT^Nt^A*) indicates that the strain has in its ancestry one or more self-fertile dikaryotic strain (*i.e.*, *N. tetrasperma*-like).

*N. crassa* / *N. tetrasperma* hybrid strain: *C4,T4 a* (FGSC 1778; Metzenberg and Ahlgren 1969) was used as a bridging strain to initiate the introgression of *N. crassa* genes into *N. tetrasperma*. Crosses of *C4,T4a* with opposite mating type strains of *N. crassa* and *N. tetrasperma* strains can produce small numbers of viable progeny.

### Ascospore harvests

Ordinarily ascospores can be harvested by simply washing the lids of the Petri plates in which the crosses are made with 1.5 ml water, concentrating the ascospore suspension to 0.1-0.5 ml, and then counting an aliquot in a hemocytometer. This is simple enough to do and one can obtain adequate ascospore numbers. However, we noticed that many round as well as some spindle-shaped ascospores remained adhered to the outside of perithecia, or were deposited close by on the agar, especially in the crosses involving the B/S1 strains and in *N. tetrasperma*. This suggested that round ascospores might suffer impaired dispersal relative to the spindle-shaped ones, and the former might also interfere with dispersal of the latter, possibly by clogging the perithecial exit pore (ostiole). In which case, scoring only the lid-harvested ascospores might underestimate MSUD strength. The results in Tables 1, 2, and S4 represent “total harvests”. They were obtained by combining the “lid harvests” with ascospores harvested by scraping the perithecia, mycelia, and ascospores from the agar surface of the cross plate with a rubber policeman or spatula into 2 ml water, transferring the suspension to a 2 ml tube, spinning for 60 sec in a table top centrifuge to pellet the ascospores, and removing the supernatant with debris. The ascospore pellet was re-suspended in 0.1-0.5 ml water and an aliquot counted in a hemocytometer. Tables 1 and S4 present the results from the total harvests in crosses heterozygous or homozygous for the ::*r3* and ::*r1* transgenes, whereas Tables S5 and S6 represent the corresponding lid harvests. It is apparent that the difference between the two harvests was not as pronounced in the OR background. Liu *et al.* (2017) have suggested that A-to-I mRNA editing “corrects” conserved premature stop codons in genes related to ascosporogenesis and ascospore discharge, therefore the altered ascospore dispersal might reflect editing differences in B/S1 and *N. tetrasperma* relative to OR (see Discussion section).

**Table 2:** MSUD-induced round ascospore fractions in *N. tetrasperma*

Serial No.	Cross	N (x10^5^)	Round ascospores (%)
1	85 *a* x 85 *A*	1.8	0
2	::*r3^Nt^ a* x 85 *A*	0.4	5.1 ± 0.8
3	85 *a* x ::*r3^Nt^ A*	0.4	7.1 ± 0.9
4 (i)	::*r3^Nt^ a* x ::*r3^Nt^ A*	0.2	1.8 ± 0.4
4 (ii)	::*r3^Nt^ a* x ::r3^Nt^ A*	0.5	11.3
5	::*r3^Nt^ a* x *WT^Nt^ A*	0.8	21.5 ± 3.9
6	*Sad-2^Nt^ a* x 85 *A*	1.3	0
7	*Sad-2^Nt^ a* x ::*r3^Nt^ A*	Infertile	—
8	85 *a* x *Sad-2^Nt^ A*	1.3	0
9	::*r3^Nt^ a* x *Sad-2^Nt^ A*	0.3	1.9 ± 0.5
10	*Sad-2^Nt^ a* x *Sad-2^Nt^ A*	Infertile	—

N = number of ascospores harvested. Round ascospore fractions are given as mean percentage ± SEM from four technical replicates of each cross. Harvests were made on days 11-20. * This cross used strain CD #1 as the putative ::*r3^Nt^A* parent, but the CD #1 strain turned out to be a dikaryon, see text.

### Construction of MSUD testers in the B/S1 background

A cross was made between the wild-isolated strains Bichpuri-1 *a* and Spurger-3 *A* (B *a* x S *A*) and the f_1_ progeny were used to make four f_1_ *a* x f_1_ *A* sib-pair crosses that initiated four recombinant inbred lines (Figure S2A). Within a line, in each generation sibling progeny of opposite mating types were crossed to produce the next generation (*i.e.*, sibling f_1_ *a* x f_1_ *A* to produce the f_2_, then sibling f_2_ *a* x f_2_ *A* to produce the f_3_, etc). A recessive sterility-causing mutation became homozygous in the f_4_ and f_3_ generation of, respectively, lines 2 and 4 (C. Usha and D. P. Kasbekar, unpublished results), but we were able to reach the f_10_ generation in lines 1 and 3. A *mat A* and *mat a* strain pair of the f_10_ generation of line 1 (specifically, segregants #1 and #3) are referred to henceforth as B/S1 *A* and B/S1 *a* (Figure S2A). A similar schematic diagram illustrating the early crosses (upto the f_8_ generation) was presented in Nagasowjanya *et al.* (2013).

The *mus-51* gene in the B/S1 background was mutated by RIP (Selker 1990). Strains mutant in *mus-51* are defective for non-homologous end joining, consequently, any transforming DNA can integrate only via homologous recombination (Ninomiya *et al.* 2004). A DNA construct bearing a 1683 bp *mus-51* segment (-205 to 1478 bp of the 2046 bp MUS-51 ORF) and the hygromycin-resistance (*hph*) cassette (Carroll *et al.* 1994) was transformed by electroporation into B/S1 *A* conidia, and ectopic integration of the transforming DNA created the transgenic *Dp(mus-51)* duplication. The *Dp(mus-51) A* primary transformant was crossed to B/S1 *a* and the progeny were used to perform a *Dp(mus-51)*-homozygous cross. Of 40 progeny examined from late harvested ascospores, one *mat a* progeny (#24) was found to contain several RIP-induced mutations in the endogenous *mus-51* gene, including in-frame stop codons (Genbank accession number KM025239), and it was crossed with the B/S1 *A* strain to segregate out the *Dp(mus-51)* transgene and obtain the B/S1 *mus-51 A* #8, B/S1 *mus-51 a* #3 strains in the progeny.

Transformation of B/S1 *mus-51A* conidia was used to make the *tester^B/S1^* strains, namely, ::*r1^B/S1^* and ::*r3^B/S1^*, which are analogous to the OR-derived ::*r1^OR^* and ::*r3^OR^* testers of Samarajeewa *et al.* (2014). A 2532 bp DNA fragment (called *r^ef^*) was amplified by PCR from the 3′ end of the OR-derived *r^+^* gene and joined to the *hph* cassette by double-joint PCR (Yu *et al.* 2004) to create a 4.1 kb *r^ef^-hph* fusion construct. The fusion construct’s flanking sequences were derived from the B/S1 background to enable it to be precisely inserted by homologous recombination into the same genomic sites as the ::*r1^OR^* and ::*r3^OR^* testers (Figure S2B). The DNA constructs were transformed by electroporation into the B/S1 *mus-51 A* strain, and transformants were selected on hygromycin-medium. The potentially heterokaryotic primary transformants were crossed with B/S1 *a* to segregate out the *mus-51* mutation and obtain a ::*r3^B/S1^ A* homokaryon from whose cross with B/S1 *a* we obtained the segregants #15 and #24 (of genotype ::*r3^B/S1^ a*) and #4 and #16 (of genotype ::*r3^B/S1^A*). In a like manner we obtained an ::*r1^B/S1^ A* homokaryon that was crossed with B/S1 *a* to obtain the segregant #4 (of genotype ::*r1^B/S1^ a*) and segregant #1 (of genotype ::*r1^B/S1^ A*). Between derivation of the B/S1 *A* and *a* strains and the ::*r^B/S1^ A* and *a* strains five additional backcrosses were done to B/S1, thus increasing the chance that a ::*r^B/S1^ A* x ::*r^B/S1^ a* cross is further reduced in heterozygosity compared to a B/S1 *A* x B/S1 *a* cross.

Strains B/S1 *A* #1 (FGSC 26446), B/S1 *a* #3 (FGSC 26445), B/S1 *mus-51 A* #8 (FGSC 26444), B/S1 *mus-51 a* #3 (FGSC 26443), VIIL ::*r1^B/S1^-hph A* #1 (FGSC 26442), VIIL ::*r1^B/S1^-hph a* #4 (FGSC 26441), VIIL ::*r3^B/S1^-hph A* #16 (FGSC 26440), and VIIL ::*r3^B/S1^-hph a* #15 (FGSC 26439) have been deposited in the FGSC, with the accession numbers indicated in parenthesis.

### Derivation of the ::r3^Nt^ and WT^Nt^A strains

Strain ISU 3119 (genotype *VIIL*::*r3 A*) was crossed with the *N. crassa* / *N. tetrasperma* hybrid bridging strain C4T4 *a* and an ::*r3^1C^A* progeny was back-crossed with C4T4 *a* to produce an ::*r3^2C^ a* progeny, that was crossed with 85*A*, and an ::*r3^185^a* progeny was back-crossed with 85 *A*. (Superscript “1C^”^ indicates progeny from the first cross with C4T4 *a*, “2C^”^ indicates progeny from the second cross with C4T4 *a*, and “n85” indicates progeny from the nth backcrosses with strain 85.) From the cross ::*r3^185^a* x 85 *A* we obtained several self-fertile progeny bearing the ::*r3* transgene, *viz*., 1R, 2R, 6R, 8R, 10R, 13R, 15R, 17R, and 20R. Self-cross of these self-fertile strains produced mostly four-spored asci, including several with round ascospores (% round ascospores, respectively, 90, 5, 5, 45, 90, 1, 2, 55, and 1). The crosses whose results are summarized in Table 2 were made with self-sterile conidial derivatives (CD) obtained from these self-fertile strains. The *VIIL*::*r3^Nt^ a* strain used was CD #4 derived from 10R, and *VIIL*::*r3^Nt^ A* was CD #5 derived from 1R. The ::*r3^Nt^ a* strain was deposited in the FGSC with the accession number FGSC 26489.

The *WT^Nt^A* strain in Table 2 was CD #5 derived from 6R, and it contains the non-transgenic nucleus of the 6R dikaryon. It was used as an additional “wild type” control.

CD #1 derived from 13R turned out to be a self-sterile [::*r3^Nt^A* + *WT^Nt^A*] dikaryon whose identification and possible provenance are described in the Results and Discussion sections.

The oligonucleotide primers dhphF and dhphR (Table S2) were used to PCR amplify a 1.5 kb segment from genomic DNA of *N. crassa* and *N. tetrasperma* strains containing the chromosome 7 transgene ::*r3*. Strains bearing a transgene-free chromosome 7 were detected by PCR amplification of a 2.5 kb amplicon with the primers (Table S2) r^Nt^WT-F and either r^Nt^WT-OR-R (for OR-derived DNA) or r^Nt^WT-85-R (for strain 85-derived DNA).

### Derivation of Sad-2^Nt^ strains

The construction of the *Sad-2^Nt^A* and *Sad-2^Nt^a* strains is schematically outlined in Figure S1. The bent arrows in the figure indicate identification of the *Sad-2* progeny by PCR. The primers sad-2spc-F and sad-2spc-R (Table S2) can amplify a 1 kb segment in PCR using genomic DNA of strains bearing the *Sad-2* (*RIP32*) allele as template, whereas primers sad-2+spc-F and sad-2+spc-R (Table S2) can amplify a 619 bp amplicon in PCR with genomic DNA of *N. crassa* and *N. tetrasperma* strains containing the wild type *sad-2^+^* allele. Briefly, from the cross of the *N. crassa* RLM 30-12 strain (genotype *Sad-2* (*RIP32*) *A*) with *C4,T4 a* we identified a *Sad-2^1C^ a* progeny by PCR, and from the cross *Sad-2^1C^ a* x 85*A* we obtained a *Sad-2^185^ a* segregant and used it to initiate a series of backcrosses with the 85*A* or 85 *a* strains. After six successive backcrosses we obtained a self-sterile [*Sad-2^685^ A* + *Sad-2^685^ a*] dikaryon that was productive in crosses with both 85 *A* and 85 *a*. From the dikaryon’s cross with 85 *a* we obtained S12, a self-fertile dikaryon of presumed genotype [*Sad-2^785^a* + *sad-2^+^ A*]. From the self-cross of S12 we obtained the [*Sad-2* + *sad-2*^+^] dikaryons 1S12 and 3S12, and, from the self-cross of 1S12 we obtained the [*Sad-2* + *sad-2*^+^] self-fertile dikaryons 1(1S12), 3(1S12), 4(1S12), 9(1S12). The *Sad-2^Nt^A* strain used in Table 2 is a *mat A* conidial derivative from 3(1S12). To derive the *Sad-2^Nt^a* strain we obtained one *Sad-2 a* homokaryotic progeny (#20) from the cross of the self-sterile [*Sad-2^685^ A* + *Sad-2^685^ a*] dikaryon with 85 *A*. This cross yielded mostly self-fertile progeny, including one called S1, from which we obtained the *Sad-2^Nt^ a* strain as a conidial derivative, and it is deposited in the FGSC with accession number FGSC 26490.

### Whole genome sequence analysis of Neurospora strains

DNA samples were sequenced at Fasteris, Switzerland, on Illumina HiSeq 4500 platform with paired end reads and read length of 150 bp. Quality and statistics of the raw reads were analyzed using FastQC (Version 0.11.5). Illumina adapters from the paired reads were removed using trimmomatics (version 0.36) (Bolger *et al.* 2014). Preprocessed reads were aligned to the *N. crassa* OR74A reference genome (version NC12, release 38, ensembl) using bowtie2 (version 2.3.4) with end-to-end parameter (Langmead and Salzberg 2012). All samples were observed to have more than 80% alignment rate and at least ∼8 million aligned reads (Table S3). Duplicated reads in the alignment file were removed using picardtools (version 2.17.4). Variant calling or genotyping of the thirteen samples (NC1 to NC13) was performed using Gatk HaplotypeCaller (version 4.0.0.0) (DePristo *et al.* 2011; Van der Auwera *et al.* 2013). We filtered out SNPs with QD (Quality by depth) < 20. Genotype comparison among the NC1 to NC13 samples were performed using custom R script (version 3.4.3). The whole genome sequence data for the NC1 to NC13 strains are available from the National Centre for Biotechnology Information Sequence Read Archive under the accession number SRP149022.

### Data availability statement

Strains and plasmids are available upon request. We affirm that all data necessary for confirming the conclusions of the article are present within the article, figures, and tables. Nucleotide sequence data are available at GenBank under accession numbers KM025239; MG009253; MG009254; MG009255; MG009256; MG017489; MG017490; MG017491; MG017492; MK392332 and MK392333. Whole genome sequence data for 15 *N. crassa* strains are available at the National Centre for Biotechnology Information Sequence Read Archive under accession number SRP149022. Supplementary Tables S1-S6 and Figures S1, S2 are placed in figshare.com with doi: 10.6084/m9.figshare.7570934. Table S1 lists the Neurospora strains used, Table S2 lists the oligonucleotide primers, Table S3 summarizes the Illumina whole genome sequencing statistics, Table S4 presents the round ascospore fractions from crosses involving the ::r1 strains, Table S5 presents the round ascospore fractions in the lid harvests from crosses involving the ::r3 strains, Table S6 presents the round ascospore fractions in the lid harvests from crosses involving the ::r1 strains, Figure S1 is a schematic depiction of the derivation of the *Sad-2^Nt^* strains, and Figure S2 is a schematic depiction of the construction and use of the ::*r^B/S1^* tester strains.

## Results

### Genome heterozygosity in the near-isogenic ::r3^B/S1^ x B/S1 crosses

Previous work had revealed that only ∼10% of wild-isolated *N. crassa* strains (W) examined in crosses with the OR-derived MSUD testers showed an OR-type “efficient MSUD” phenotype (Ramakrishnan *et al.* 2011). If the hypothesis that MSUD is suppressed by genome sequence heterozygosity in *tester^OR^* x W crosses is correct, then crosses isogenic for a wild-derived non-OR type genome (*i.e.*, *tester^W^* x W) should exhibit efficient MSUD as in the OR background. To test this, we generated the novel near-isogenic strains B/S1 *A* and B/S1 *a* (see Materials and methods) from the Sad-type wild strains Bichpuri-1 *a* and Spurger-3 *A* and tested MSUD efficiency in this novel background.

In the series of 10 successive sib-pair cross performed to create the B/S1 strains, the residual heterozygosity in each cross is half the value in the previous cross (100%, 50%, 25%, …, 0.4%, 0.2%, 0.1%). Hence the B/S1 *A* x B/S1 *a* cross is expected to be heterozygous in only ∼0.1% (*i.e.*, ∼44 kbp) of the *mat*-unlinked genome fraction. To verify this, we used Illumina whole genome sequencing to identify 8784 SNPs between the B/S1 *A* and B/S1 *a* genomes. This number excluded the SNPs within the *mat* locus, in which the *mat A* and *mat a* idiomorph sequences are very dissimilar and seemingly unrelated. The Bichpuri-1 *a* and Spurger-3 *A* genome sequences are not available, therefore we could not determine the percentage loss of heterozygosity (LOH) or map the LOH boundaries. Instead, of the 8784 SNPs, 7961 (90.6%) were found to be in a 570 kb *mat*-linked genome segment (see below), and in it the SNP density was 14 SNPs /kb (7961 SNPs/ 570 kb). Of the remaining SNPs, 91 were elsewhere on chromosome I, and 732 were distributed on the other chromosomes (chromosome 2 - 387; 3 - 49; 4 - 104; 5 - 113; 6 - 20; and 7 - 59). Thus, the SNP density in the *mat*-unlinked genome fraction was 0.02 SNPs/ kb (823 SNPs/ 39894 kb). The ratio 0.02/14 (0.14%) of SNP density in the *mat*-unlinked and *mat*-linked genome fractions agrees with the 0.1% estimate made above.

**Figure 1:**
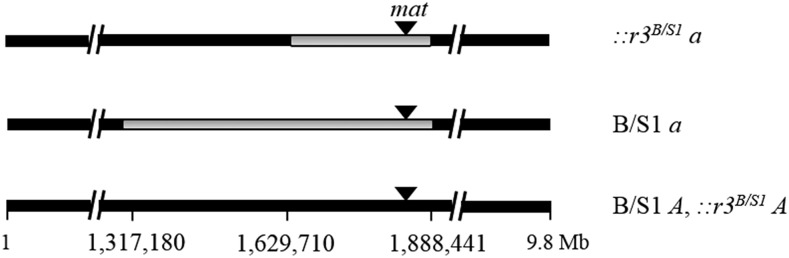
B/S1 crosses are heterozygous in *mat*-linked region. Chromosome 1 is 9.8 Mb long and its nucleotides are numbered as per the OR genome sequence (ID- CM002236). In the B/S1 *a* and ::*r3^B/S1^ a* strains the *mat a* locus and flanking sequences are derived from the Bichpuri-1 *a* strain and shown in gray, whereas in the B/S1 *A* and ::*r3^B/S1^ A* strains the corresponding sequences, shown in black, are derived from Spurger-3 *A*. In B/S1 *a* and ::*r3^B/S1^ a* sequences distal (leftward) to, respectively, nucleotides corresponding to positions 1317180 and 1629710 in the OR genome sequence, and proximal (rightward) to the nucleotide corresponding to position 1888441, are identical to those in the B/S1 *A* sequence. Thus, the crosses ::*r3^B/S1^ a* x B/S1 *A* and ::*r3^B/S1^ a* x ::*r3^B/S1^ A* are heterozygous for a ∼258,731 bp *mat*-linked segment, whereas the crosses B/S1 *a* x B/S1 *A* and B/S1 *a* x ::*r3^B/S1^A* are heterozygous for a ∼570,329 bp *mat*-linked segment. Overall, the ::*r3^B/S1^a* x B/S1 *A* and B/S1 *a* x ::*r3^B/S1^A* crosses are heterozygous for, respectively, ∼303 kbp (0.7%) and ∼614 kbp (1.4%) of the genome (see text). Sequence accession numbers are MG009253 and MG017489 (Spurger), MG009254, MG017490 and MK392333 (B/S1 *A*); MK392332 (B/S1 *a*); MG009255 and MG017491 (::*r3^B/S1^a*), and MG009256 and MG017492 (Bichpuri).

As described in the Materials and Methods, construction of the ::*r^B/S1^ A* and *a* strains entailed doing five additional backcrosses to the B/S1 background, that potentially could further reduce heterozygosity in ::*r1^B/S1^ A* x ::*r1^B/S1^ a* crosses relative to that in B/S1 *A* x B/S1 *a*. We identified a series of PCR-based Bichpuri-1 *vs.* Spurger-3 RFLP markers in the chromosome 1 *mat*-distal (d) and *mat*-proximal (p) regions and determined whether they were homozygous or heterozygous (designated, respectively, with subscripts i and j) in a ::*r3^B/S1^ A* x ::*r3^B/S1^ a*, B/S1 *a* x B/S1 *A*, or B/S1 *a* x ::*r3^B/S1^A* cross, until the d_i_ - d_j_ and p_i_ - p_j_ intervals were small enough to be sequenced from the ::*r3^B/S1^ A*, ::*r3^B/S1^ a*, B/S1 *A*, and B/S1 *a* genomic DNA. Alignment of the sequences revealed that the ::*r3^B/S1^ a* x ::*r3^B/S1^ A* and ::*r3^B/S1^ a* x B/S1 *A* crosses were heterozygous for an ∼258,731 bp *mat*-linked genome segment, whereas the B/S1*a* x ::*r3^B/S1^A* and B/S1*a* x B/S1 *A* crosses were heterozygous for an ∼570,329 bp *mat*-linked segment (Figure 1, the “∼” indicates the presumed differences in nucleotide sequence between the OR and Bichpuri-1 or Spurger-3 genomes). These segments contain, respectively, 80 and 151 genes. To these values if we add the ∼44 kbp corresponding to heterozygosity in the *mat*-unlinked genome fraction, then the first two crosses are heterozygous for ∼303 kbp (0.7%), and the latter two are heterozygous for ∼614 kbp (1.4%), of the genome. The B/S1 x ::*r1^B/S1^* crosses also are heterozygous for no more than 1.4% of the genome. The ∼570 kb *mat*-linked segment does not include the chromosome 1 gene *sad-1* (NCU 02178, nucleotides 969499 - 974473).

### MSUD efficiency differs in the N. crassa OR and B/S1 backgrounds

Model 1 (see Introduction) predicts that MSUD would be more efficient in the B/S1 x ::*r^B/S1^* crosses than in OR x ::*r^B/S1^* or B/S1 x ::*r^OR^*, since the latter crosses are surely more heterozygous. The results summarized in Tables 1 and S4 allow us to reject this model. As can be seen in the Tables, four control crosses that did not contain any ::*r* transgene (OR *A* x OR *a*, B/S1 *A* x B/S1 *a*, OR *A* x B/S1 *a*, and OR *a* x B/S1 *A*) did not produce any round ascospores, although the B/S1 *A* x B/S1 *a* cross was much less productive than OR *A* x OR *a* and the two OR x B/S1 crosses. The ::*r3^OR^* x OR crosses produced > 96% round ascospores (Figure S2C, panel i), while the homozygous ::*r3^OR^ a* x ::*r3^OR^ A* cross yielded 1% round ascospores (Table 1, Figure S2C, panel ii). This confirmed the efficient MSUD characteristic of the OR background. The two ::*r3^B/S1^* x ::*r3^OR^* crosses produced < 4% round ascospores, showing that the MSUD machinery recognizes the ::*r3^B/S1^* and ::*r3^OR^* transgenes as alleles (paired). The ::*r3^OR^* x B/S1 and ::*r3^B/S1^* x OR crosses produced 25- 64% round ascospores (see Figure S2, panel iii for an example), consistent with the results of Ramakrishnan *et al.* (2011) showing inefficient MSUD in crosses of the OR-derived testers with the progenitors of the B/S1 strains, namely, Bichpuri-1 and Spurger-3. Significantly, the ::*r3^B/S1^* x B/S1 crosses produced < 24% round ascospores, which was inconsistent with model 1. Additionally, the homozygous ::*r3^B/S1^ a* x ::*r3^B/S1^ A* cross produced ∼1.5 fold more round ascospores than the ::*r3^B/S1^* x ::*r3^OR^* crosses. Crosses with the ::*r1^OR^* and ::*r1^B/S1^* testers gave similar results (Table S4). Overall, the results show MSUD is less efficient in the B/S1 than the OR background, and there may be an increase in inappropriate silencing of paired genes in the B/S1 background. The ::*r^OR^* x B/S1 and ::*r^B/S1^* x OR crosses in Tables 1 and S4 showed that the “efficient MSUD” phenotype of OR was recessive to the “inefficient MSUD” phenotype of B/S1.

**Figure 2:**
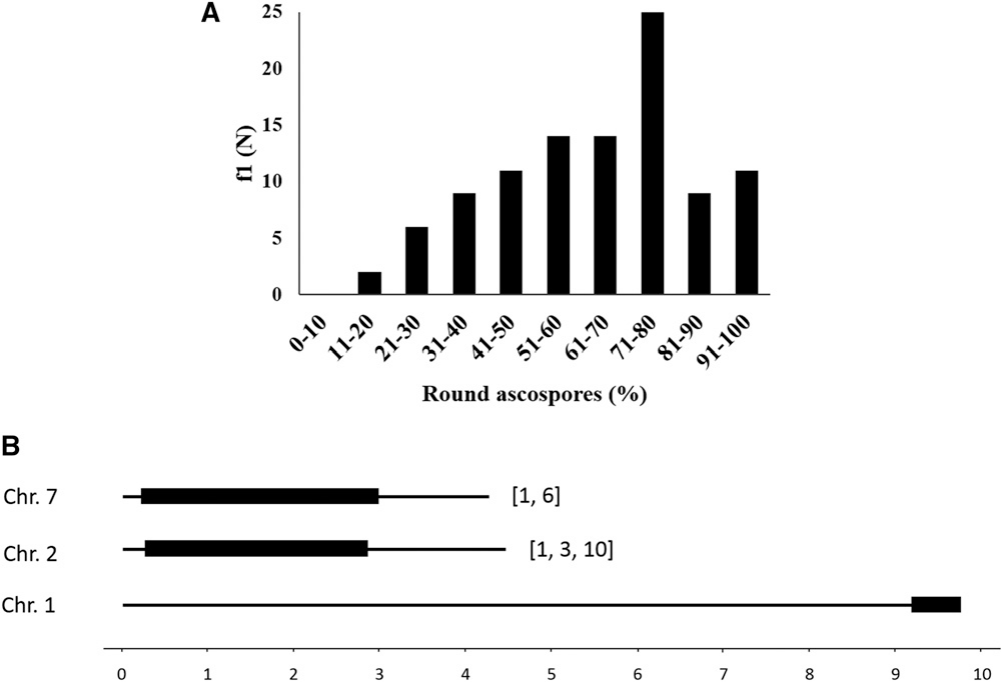
Analysis of f_1_ progeny from B/S1 *A* x OR *a*. (A) MSUD efficiency was measured as the fraction of round ascospores produced in crosses of 101 f_1_ progeny from B/S1 *A* x OR *a* with ::*r3^OR^* strains of opposite mating type, and 11 f_1_ progeny whose crosses produced >90 round ascospores were deemed to have inherited the efficient MSUD phenotype of the OR *a* parent. (B) Illumina whole genome sequencing revealed an OR-derived chromosome 1 segment (coordinates 9.5-9.8 Mb) was conserved in all the 11 OR-type f_1_ progeny. Additionally, a majority (8 and 9, respectively,) of the OR-type f_1_ progeny also contained OR-derived segments of chromosome 2 (0.3-2.8 Mb) and 7 (0.2-3.1 Mb). The box brackets indicate the OR-type progeny from which the corresponding segments were absent.

Next, we crossed 101 f_1_ progeny from B/S1 x OR with ::*r3^OR^* strains of the opposite mating type. Eleven crosses produced > 90% round ascospores (Figure 2A), suggesting that only ∼11% of the f_1_ progeny had inherited the OR-type phenotype. Illumina whole genome sequences of the 11 OR-type strains, and of two control non-OR-types (whose crosses produced 40% and 29% round ascospores), revealed that all the OR-type progeny shared a ∼300 kb OR-derived chromosome 1 segment (Figure 2B), which was absent from the two non-OR-type control progeny. Further, a majority (8 and 9, respectively,) of the OR-type progeny also contained OR-derived segments of chromosomes 2 and 7. The segments might contain genes that contribute to the OR-type phenotype.

### MSUD is inefficient in N. tetrasperma 85

As a second test of the hypothesis that non-OR strains commonly exhibit inefficient MSUD we examined comparable crosses made in *N. tetrasperma* strain 85 (Table 2). Again, control crosses not containing the ::*r3^Nt^* transgene (85 *A* x 85 *a*, 85 *A* x *Sad-2^Nt^ a*, and *Sad-2^Nt^ A* x 85 *a*) did not produce any round ascospores, and the *Sad-2^Nt^ A* x *Sad-2^Nt^ a* cross was infertile like its *N. crassa* counterpart. The ::*r3^Nt^* x 85 crosses produced ∼5–7% round ascospores, which was far less than the >96% seen in the cross ::*r3^OR^* x OR. The *WT^Nt^A* strain, which contains a transgene-free chromosome 7, was used as an additional “wild-type” control and the ::*r3^Nt^a* x *WT^Nt^A* cross produced 21.5% round ascospores. The *WT^Nt^A* strain was a conidial derivative from the 6R dikaryon obtained during the introgression of the ::*r3^Nt^* transgene from *N. crassa* OR into *N. tetrasperma* 85 (see Materials and methods).

One ::*r3^Nt^A* x ::*r3^Nt^ a* cross and the cross *Sad-2^Nt^ A* x ::*r3^Nt^ a* yielded < 2% round ascospores, which suggested, respectively, that MSUD does not occur in a transgene-homozygous cross, and that it is suppressed by *Sad-2^Nt^*. A second putative ::*r3^Nt^A* x ::*r3^Nt^ a* cross, made using the strain CD #1 as the ::*r3^Nt^A* parent, yielded 11.3% round ascospores, which was unexpectedly high for a presumed ::*r3^Nt^*–homozygous cross, and it was explored further as described in the next section.

The crosses *Dp(EB4)^Nt^A* x 85 *a* and *Dp(IBj5)^Nt^a* x 85 *A* represent *Dp*-heterozygous crosses in the *N. tetrasperma* 85 background. Significantly, neither cross showed an obvious barren phenotype, and they were only quantitatively less productive than the control *T(EB4)^Nt^a* x 85 *A* and *T(IBj5)^Nt^a* x 85 *A* crosses. *Dp(EB4)^Nt^A* x 85 *a* and *T(EB4)^Nt^a* x 85 *A* produced, respectively, 8.2 × 10^5^ and 12.4 × 10^5^ ascospores, and *Dp(IBj5)^Nt^a* x 85 *A* and *T(IBj5)^Nt^a* x 85 *A* produced 1.3 × 10^5^ and 7.1 × 10^5^ ascospores. The *Sad-2^Nt^* x *E* and *E* x *Sad-2^+^* crosses did not appear to differ in ascus development (data not shown).

### A self-sterile dikaryon recovered from a self-fertile strain

The CD #1 strain was used as the ::*r3^Nt^A* parent in the second of the two putative ::*r3^Nt^A* x ::*r3^Nt^ a* crosses, but yielded an unexpectedly high fraction of round ascospores (see the previous section). CD #1 was a self-sterile conidial derivative obtained from the self-fertile strain 13R (see Materials and Methods), and so we considered the possibility that it was in fact an [(::*r3^Nt^A*) + (*+ A*)] dikaryon. In which case, its cross with the ::*r3^Nt^ a* strain would represent two crosses, one of genotype (::*r3^Nt^A*) x ::*r3^Nt^ a*, and the other of genotype (*+ A*) x ::*r3^Nt^ a*, and the latter would produce most of the round ascospores. PCR with CD #1 genomic DNA and primers specific for either a transgene-bearing or transgene-free chromosome 7 revealed that such indeed was the case. We discuss below the likely provenance of a dikaryotic [(::*r3^Nt^A*) + (*+ A*)] conidial derivative from a self-fertile strain.

## Discussion

### Efficient MSUD is not necessarily the norm in Neurospora

MSUD in the *N. crassa* B/S1 and *N. tetrasperma* 85 backgrounds was less efficient than in the *N. crassa* OR background. In OR, MSUD in crosses heterozygous or homozygous for an ::*r* transgene was all-or-none (*i.e.*, > 97% *vs.* < 1% round ascospores), whereas in B/S1 and 85 the results were not as clear-cut, and were, respectively, 24% *vs.* 6%, and 7% *vs.* 1.4%. MSUD efficiency in B/S1 was comparable to that in OR crosses deficient for *sad-6^+^*, which encodes a Rad54-like SNF2 helicase-related protein (Samarajeewa *et al.* 2014), or for *cbp-20^+^* and *cbp-80^+^*, that encode the cap-binding complex (CBC) proteins associated with the 5′ cap of eukaryotic mRNA transcripts, which interacts with the Argonaute protein SMS-2 in the meiotic silencing complex (MSC) (Decker *et al.* 2017). Decker *et al.* (2017) have speculated that the absence of the CBC makes it harder for the MSC to recognize target mRNAs, allowing some to reach the translational machinery and thus reduces MSUD efficiency. Additionally, the inappropriate silencing of paired genes was apparently increased in B/S1. Thus, efficient MSUD, though characteristic of OR, is not necessarily the norm in *N. crassa*. The OR phenotype was recessive to the B/S1 phenotype in OR x B/S1 crosses. Possibly, extrinsic or intrinsic cues that modulate the MSUD response in B/S1 and *N. tetrasperma* 85 are absent from OR, thus making the response all-or-none. Alternatively, the B/S1 background might be null or hypomorphic for a locus encoding a protein, non-coding RNA, or small RNA that enhances the efficiency or half-life of masiRNA in the OR background. The presence of both OR- and B/S1-derived high and low efficiency alleles in an OR x B/S1 cross might result in MSUD of intermediate efficiency. The use of OR strains for genetic studies probably fortuitously facilitated (1) the discovery of MSUD and its semi-dominant *Sad* suppressors; (2) the discovery of ascus-dominant mutants such as *Ban*, *Dip-1*, *Pk*^D^, and *R*; and (3) the identification of *Dp* strains via their barrenness in crosses. Efficient MSUD in OR enabled Turner and Perkins (1982) to recover normal-sequence progeny from a cross between two chromosome 1 inversions (Kasbekar 2006).

The 2532 bp OR-derived *r* gene segment used to construct the ::*r* transgenes in the B/S1 strains differed by 16 SNPs from the corresponding B/S1 sequence, thus creating a ∼0.63% heterozygosity in ::*r3^B/S1^* x B/S1 crosses between the source of the masiRNA and their target mRNAs. We think this is too little to account for the reduced MSUD seen in the B/S1 background for the following reason. In Table 1, crosses 10, 11, 14, and 15, one *r^+^* allele targeted for MSUD is from OR and the other is from B/S1, and the round ascospore fractions ranged between 25–56%. Additionally, one OR-type f1 progeny from OR *a* x B/S1 *A* contained the B/S1 *r^+^* allele, and its cross with the ::*r3^OR^* tester gave 91% round ascospores. The 25–91% range in these crosses must have causes extrinsic to the sequence difference between the masiRNA and its target mRNAs. Similar results were obtained with the ::*r1* transgene (Table S4). Hence the difference in round ascospore fraction in ::*r^OR^* x OR *vs.* ::*r^B/S1^* x B/S1 (>95% *vs.* <25%) is unlikely to be wholly intrinsic to the sequence difference in the latter cross. Moreover, the *r* locus was outside the ∼300 kb OR-derived chromosome 1 segment found conserved in all 11 f_1_ progeny that showed the OR phenotype in crosses with the ::*r^OR^* testers. However, a conclusive demonstration would require re-doing the ::*r^B/S1^* x B/S1 crosses with a B/S1-derived ::*r* transgene.

### Genetic difference between OR and B/S1 strains

Of 101 f_1_ progeny examined from B/S1 x OR, 11 had inherited the OR-type phenotype and all of them contained an OR-derived ∼300 kb chromosome 1 segment (Figure 2B). The segment was absent from two control non-OR-type f_1_ progeny. Two non-mutually exclusive interpretations are compatible with these results. One, since fewer than 50% of the f_1_ progeny showed the OR-type phenotype, the OR-derived chromosome 1 segment might be necessary but not sufficient for efficient MSUD. In other words, the phenotype is complex. Also since a majority (8 or 9) of the OR-type progeny also contained OR-derived segments of chromosomes 2 and 7 (Figure 2B), the latter segments might contain additional genes that contribute to the OR phenotype. The other interpretation posits that the OR-derived chromosome 1 segment is both necessary and (at least largely) sufficient for the OR phenotype, *i.e.*, the phenotype is not complex, but a transmission ratio distortion (TRD) skews its inheritance into fewer than 50% f_1_ progeny. A preliminary experiment comparing the fraction of f_1_ progeny containing the OR *vs.* B/S1 “allele” revealed a 14:39 ratio, suggesting a TRD disfavoring the OR-derived segment (D. A. Giri and D. P. Kasbekar, unpublished results). We do not yet know whether the TRD and the OR-type phenotype are mechanistically related or are merely genetically linked.

The chromosome 7 candidate segment includes the *sad-5* gene (1,640,838-1,642,206) Crosses null for *sad-5* are completely deficient in MSUD (Hammond *et al.* 2013a). However, it is premature at this time to focus on individual genes, since each candidate segment contains hundreds of other loci. It will be necessary to first narrow down the candidate segments, say by using OR and B/S1 parents with flanking auxotrophic markers, select for prototrophic crossover progeny, and examine the OR-type among them to localize the relevant genes.

Some of the MSUD efficiency difference between the OR, B/S1, and *N. tetrasperma* 85 backgrounds might arise from differences in sexual-stage-specific A-to-I mRNA editing of MSUD gene transcripts. The editing converts specific adenosine residues (A) in mRNA to inosine (I), and since I is recognized as guanosine (G), therefore the effect is similar to an A-to-G substitution. Of 24,001 editing sites found in *N. crassa* genes, 51.6% were conserved in their *N. tetrasperma* orthologs (Liu *et al.* 2016; 2017). In OR, the MSUD genes *sad-1*, *-2*, *-4*, *-5*, and *-6*, *sms-2*, *dcl-1*/*sms-3*, and *qip* contain multiple non-synonymous editing sites, although *sad-3* has only one.

### Efficient MSUD suppresses Dp-mediated RIP-suppression

In general, *Dp*-heterozygous crosses in the OR background display an MSUD-dependent barren phenotype (Perkins 1997; Shiu *et al.* 2001). Most genetic studies on *Dp*s have used the OR background, hence it was widely assumed that *all* Neurospora *Dp*-heterozygous crosses are barren, notwithstanding the fact that before the advent of the *Sad* suppressors we had used wild-isolated strains (*N*) to increase progeny numbers in *Dp* x *N* crosses (Fehmer *et al.* 2001). Now, our results revealed that *T*- and *Dp*-heterozygous crosses are comparably productive in *N. tetrasperma* 85, which suggests that inefficient MSUD can increase the productivity of *Dp*-heterozygous crosses.

Further, large *Dp*s (> 300 kbp) also can act as dominant suppressors of RIP, possibly because the *Dp*s titrate out the RIP machinery (Bhat and Kasbekar 2001; Vyas *et al.* 2006; Perkins *et al.* 2007; Singh and Kasbekar 2008; Singh *et al.* 2009). *Dp*-mediated RIP suppression might be significant when *Dp*-heterozygous crosses are productive. Previous work in our laboratory had identified seven wild-isolated dominant RIP suppressor strains (Noubissi *et al.* 2001; Bhat *et al.* 2003; Vyas *et al.* 2006). It is possible that some of the RIP suppressors were *Dp* strains in inefficient MSUD backgrounds.

### Does MSUD underlie the ascus-dominant E phenotype?

We did not find any difference in ascus development in the *Sad-2^Nt^* x *E* and *E* x *Sad-2^+^* crosses, which suggested that the ascus-dominant *E* phenotype does not involve MSUD. This presupposes that the *Sad-2* mutation effectively silences *sad-2*^+^ in the 85 background. This supposition is supported by our results that ::*r3^Nt^* x 85 and *Sad-2^Nt^ A* x ::*r3^Nt^ a* produced, respectively, 5–7% and < 2% round ascospores. A more definitive experiment would be to test the *E* mutant phenotype in *N. tetrasperma* crosses homozygous for *sad-4Δ* or *sad-5Δ*. The SAD-4 and SAD-5 proteins are essential for MSUD in *N. crassa*, but unlike SAD-2, they are dispensable for ascus development (Hammond *et al.* 2013a). Jacobson (1995) had found that certain combinations of intercrosses between single mating-type conidial derivatives derived from wild-collected *N. tetrasperma* heterokaryons produced mostly eight-spored asci when one derivative was used as the female (protoperithecial) parent and mostly four-spored asci when the derivatives were reversed in the reciprocal cross, which suggests that eight-spored asci can form independently of MSUD in *N. tetrasperma*.

### Origin of the CD #1 conidial derivative

The self-sterile conidial derivative CD #1 was derived from the *N. tetrasperma* self-fertile strain 13R. Initially its genotype was assumed to be ::*r3^Nt^A*, but it turned out to be a [(::*r3^Nt^A*) + (*+ A*)] dikaryon. Therefore the 13R strain was likely a trikaryon of genotype [(::*r3^Nt^A*) + (*+ A*) + (*+ a*)] or [(::*r3^Nt^A*) + (*+ A*) + (::*r3^Nt^ a*)]. When self-crossed, these trikaryons can yield MSUD-induced round-spored asci. The trikaryotic ascospore can form if the ::*r3^Nt^* locus underwent second-division segregation, and one or more nucleus underwent an additional mitosis subsequent to the post-meiotic mitosis but prior to ascospore partitioning. Crosses involving hybrid strains obtained by introgressing *N. crassa* translocations into *N. tetrasperma* occasionally underwent such additional mitoses, and the pre-partition ascus can contain more than eight nuclei (Kasbekar and Rekha 2017), whose partitioning into four ascospores results in some containing more than two nuclei. The CD #1 strain’s genotype cautioned us to re-confirm the genotype of all strains thought to be homokaryotic in this work.
